# Influence of demographic changes on the impact of vaccination against varicella and herpes zoster in Germany – a mathematical modelling study

**DOI:** 10.1186/s12916-017-0983-5

**Published:** 2018-01-09

**Authors:** Johannes Horn, Oliver Damm, Wolfgang Greiner, Hartmut Hengel, Mirjam E. Kretzschmar, Anette Siedler, Bernhard Ultsch, Felix Weidemann, Ole Wichmann, André Karch, Rafael T. Mikolajczyk

**Affiliations:** 1 ESME - Epidemiological and Statistical Methods Research Group, Helmholtz Centre for Infection Research, Braunschweig, Germany; 2 Institue of Medical Epidemiology, Biostatistics and Informatics, Martin-Luther-University Halle-Wittenberg, Halle, Germany; 3 PhD Programme “Epidemiology” Braunschweig-Hannover, Braunschweig, Germany; 4 Department of Health Economics and Health Care Management, School of Public Health, Bielefeld University, Bielefeld, Germany; 5 Institute of Virology, Faculty of Medicine, Albert-Ludwigs-University, University Medical Center, Freiburg, Germany; 6 Julius Centre for Health Sciences & Primary Care, University Medical Centre Utrecht, Utrecht, The Netherlands; 7 Centre for Infectious Disease Control, RIVM, Bilthoven, The Netherlands; 8 Immunization Unit, Robert Koch Institute, Berlin, Germany; 9 German Center for Infection Research (DZIF), Hannover-Braunschweig site, Braunschweig, Germany; 10 Hannover Medical School, Hannover, Germany

**Keywords:** Varicella, Herpes zoster, Mathematical model, Demographic change, Vaccination

## Abstract

**Background:**

Epidemiological studies suggest that reduced exposure to varicella might lead to an increased risk for herpes zoster (HZ). Reduction of exposure to varicella is a consequence of varicella vaccination but also of demographic changes. We analyzed how the combination of vaccination programs and demographic dynamics will affect the epidemiology of varicella and HZ in Germany over the next 50 years.

**Methods:**

We used a deterministic dynamic compartmental model to assess the impact of different varicella and HZ vaccination strategies on varicella and HZ epidemiology in three demographic scenarios, namely the projected population for Germany, the projected population additionally accounting for increased immigration as observed in 2015/2016, and a stationary population.

**Results:**

Projected demographic changes alone result in an increase of annual HZ cases by 18.3% and a decrease of varicella cases by 45.7% between 1990 and 2060. Independently of the demographic scenario, varicella vaccination reduces the cumulative number of varicella cases until 2060 by approximately 70%, but also increases HZ cases by 10%. Unlike the currently licensed live attenuated HZ vaccine, the new subunit vaccine candidate might completely counteract this effect. Relative vaccine effects were consistent across all demographic scenarios.

**Conclusion:**

Demographic dynamics will be a major determinant of HZ epidemiology in the next 50 years. While stationary population models are appropriate for assessing vaccination impact, models incorporating realistic population structures allow a direct comparison to surveillance data and can thus provide additional input for immunization decision-making and resource planning.

**Electronic supplementary material:**

The online version of this article (10.1186/s12916-017-0983-5) contains supplementary material, which is available to authorized users.

## Background

Public health consequences of demographic changes on the incidence of non-communicable diseases have been discussed in the context of the so-called epidemiological transition [1–3]. However, it is usually neglected that the epidemiology of infectious diseases, which relies on dynamic transmission processes within populations, can also be affected by changing population structures and resulting changes in contact patterns. Nevertheless, in the context of childhood infections, there are some modelling studies available analyzing the effects of applying realistic population models [4–7]. Due to their increased complexity, these models usually need additional calibration data as well as simplifying assumptions, raising the question of when it may be useful to apply a realistic population model and when these additional requirements can be fulfilled.

One example of an infectious disease affected by demographic changes is herpes zoster (HZ), which is caused by the reactivation of varicella zoster virus (VZV) up to decades after initial infection, occurring commonly during childhood as varicella. Incidence and disease severity of HZ increase considerably with age so that HZ disease burden is directly affected by the ageing of a society [8]. Moreover, the risk for reactivation of VZV seems to be reduced through contact with the virus, e.g., by being exposed to children suffering from varicella (boosting hypothesis) [9, 10]. Since the frequency of contacts with children will decrease in an ageing society, overall HZ incidence is expected to increase, especially among the elderly. Varicella vaccination will further decrease VZV transmission and increase HZ incidence due to reduced boosting opportunities [11–14]. Vaccination against HZ may potentially counteract this effect. However, the currently licensed live attenuated HZ vaccine is assumed to suffer from low efficacy/effectiveness in older age groups (the persons with the highest complication and mortality rates), and from a short duration of protection [15, 16]. A new recombinant subunit vaccine candidate showed a high vaccine efficacy of 91–97% across all age groups in two recent phase III clinical trials [17, 18].

Mathematical models developed to support decision-making on immunization strategies rarely account for demographic effects. The present study evaluated how projected demographic changes in combination with vaccination strategies can affect the epidemiology of varicella and HZ in Germany and to what extent demographic changes may influence the impact of vaccination. In addition, we assessed the robustness of the predictions in the face of unexpected short-term demographic changes (as currently observed due to the increased immigration to Germany). Finally, we studied how a new HZ vaccine candidate might affect HZ epidemiology.

## Methods

### Model structure

We used an extended SEIR (susceptible, exposed, infectious, resistant) deterministic compartment model for VZV described in detail elsewhere [19]. The model (Additional file 1: Figure S1) was fitted to serological (varicella) and incidence (HZ) data from the pre- and post-varicella vaccination era in Germany, using age- and sex-specific reactivation rates for HZ [19]. Details regarding parameter values and calibration results can be found in Additional file 1: Table S1.

### Model populations

**Fig. 1:**
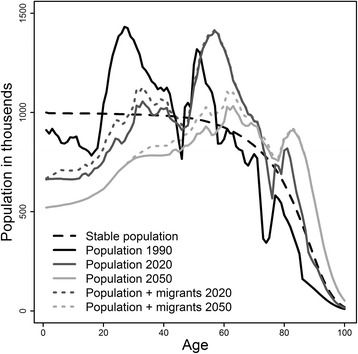
Comparison of the age distribution of the stationary population versus the projected populations from 1990 to 2060

All analyses were carried out for three different population scenarios, namely a stationary population, the projected population for Germany, and a projected population adjusted for the increased immigration as observed in 2015/2016 in Germany (projected population with increased migration) not anticipated by the population projections of the Federal Statistical Office of Germany (Destatis; https://www-genesis.destatis.de). For the stationary population scenario, we applied age- and sex-specific mortality rates for Germany from 2013 (using 1-year age groups) (Destatis) and a constant number of 1 million births per year (male:female ratio 1.05:1). This resulted in a stable population size of 80.6 million individuals, representing the German population size between 1990 (79.8 million) and 2015 (80.8 million). For the projected population, we used the age- and sex-specific population projection (including assumed future immigration and emigration patterns) provided by Destatis for the years 1990 to 2060 (Fig. 1). For simplicity, we assumed that, in this population scenario, migrants do not differ from the resident German population with respect to their varicella and HZ status; this is a reasonable assumption for individuals from other European countries having represented the majority of migrants in Germany before 2015. To reflect the short-term immigration as observed in 2015/2016, we adjusted the projected population by including an additional 1 million persons immigrating to Germany in 2015 with an ongoing influx of migrants decreasing stepwise by 100,000 per year from 2016 to 2025 (projected population with increased migration). As VZV seroprevalence differs across the nine top immigration countries and is consistently lower than in Germany, we obtained seroprevalence data for children and adults for each country as proxy for VZV seroprevalence in immigrants (Additional file 1: Table S2).

### Contact patterns

Age-specific contact patterns were implemented based on the POLYMOD survey (all contacts, irrespective of type and duration of contact) [20]. Necessary changes in contact rates over time in the projected populations caused by varying population sizes in each age group were implemented based on yearly balancing of age-specific contact rates reported in the POLYMOD survey. For this, we used the geometric mean of the total numbers of contacts of each two age groups having contact with each other. In the projected population, for example, the number of children aged < 10 years is predicted to decrease until 2060 to 58.1% of their number in 1990, whereas the number of persons aged ≥ 75 years is predicted to increase to 232.7% of their number in 1990. Consequently, the ratio between the number of persons aged < 10 years and those aged ≥ 75 years will decrease from 1.6 in 1990 to 0.4 in 2060. The ratio between the average number of contacts of children aged < 10 years with individuals aged ≥ 75 years and vice versa must then decrease by a factor of $$ \raisebox{1ex}{$1.6$}\!\left/ \!\raisebox{-1ex}{$0.4$}\right.=4 $$. As we are using the geometric mean for balancing contact rates, the average number of contacts of children aged < 10 with persons aged ≥ 75 will increase by $$ \sqrt{4}=2 $$; conversely, the average number of contacts of persons aged ≥ 75 with children aged < 10 will be halved (Additional file 1: Figure S4).

For the projected population with increased migration, we assumed that, in the first year after arrival in Germany, immigrants only have contact with other migrants plus one additional contact per day to a random person in the resident German population. Only after this first year do some immigrants leave Germany. The proportion of those remaining in Germany for each country of origin was estimated with the so called official ‘protection rates’ (proportion of accepted asylum seekers/refugees as well as individuals granted either temporary protection or deportation ban of all asylum seekers by country). For migrants remaining in Germany, we assumed the same contact patterns as for the resident German population.

### Modelling of vaccines and vaccination strategies

Varicella vaccine effectiveness was assumed to be 92% (one dose) and 95% (two doses), with an average duration of vaccine-induced protection of 40 and 80 years, respectively [19]. Vaccination coverage was set to observed rates until 2010 and assumed to be constant thereafter (86.9% for one dose at 12 months and 64.1% for two doses at 24 months; recommended age in Germany is 11–14 months for first dose, 15–23 months for second dose) [19].

For the HZ vaccination scenario, one dose HZ vaccination was assumed to start in 2015 at the age of 60 years, the most effective age at HZ vaccination with respect to reduction of HZ cases (Additional file 1: Figure S6) [19]. HZ vaccination coverage was assumed to be 20% in the base case scenario and was varied from 0 to 100% in sensitivity analyses. Age-dependent vaccine efficacy of the currently licensed live attenuated HZ vaccine was based on the results of clinical trials [21–23]. We also studied a not yet licensed recombinant subunit vaccine candidate that showed a very high vaccine efficacy across all age groups in a recent phase III study in combination with a longer duration of protection (estimated as 56 years; chapter 11 in Additional file 1) [17].

## Results

### Effect of demographic changes on the epidemiology of varicella and HZ (in absence of varicella and HZ vaccination)

**Fig. 2:**
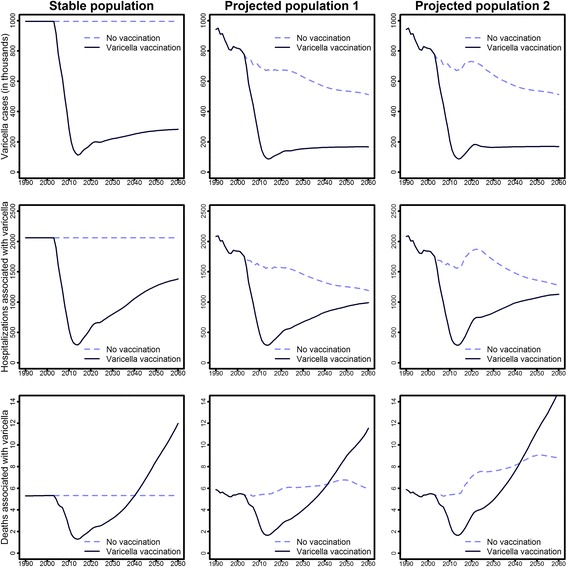
Effects of varicella vaccination on varicella cases, hospitalizations, and deaths by time and population scenario (projected population 1: predictions of the Federal Statistical Office; projected population 2: projected population with increased immigration, additionally accounting for short-term immigration)

**Fig. 3:**
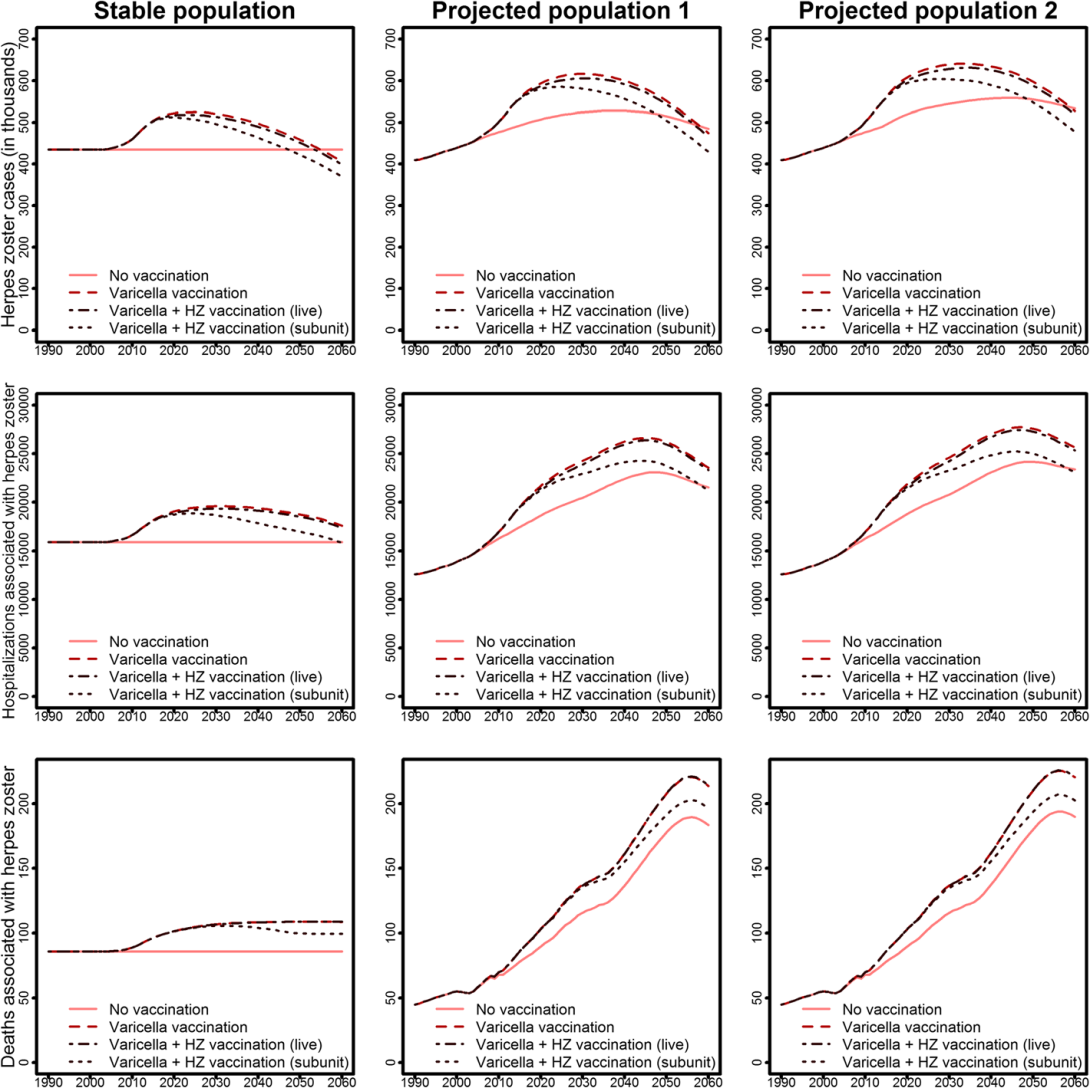
Effects of varicella and HZ vaccination on HZ cases, hospitalizations, and deaths by time and population scenario (projected population 1: predictions of the Federal Statistical Office; projected population 2: projected population with increased immigration, additionally accounting for short-term immigration)

In the projected population, the annual number of varicella cases decreased by 45.7% from 1990 until 2060 (Fig. 2, upper panel, no vaccination scenarios), while the number of HZ cases increased by 18.3% (Fig. 3, upper panel, no vaccination scenarios). This resulted in an estimated number of 500,000 cases per year for each of the two diseases in 2060. Age-specific HZ incidence rates were only affected in individuals aged ≥ 75 years (increase of 18.8% compared to 1990, data not shown).

As complication rates for varicella increase with age, the overall reduction of varicella cases was counteracted by increasing proportions of more severe varicella cases in the growing elderly population. Therefore, the decrease in varicella hospitalizations from 1990 to 2060 was smaller than for cases (–42.8%), whereas the number of deaths remained almost stable (Fig. 2). For HZ, increasing case numbers were associated with an even greater increase in hospitalizations (from 12,585 in 1990 to 21,515 in 2060) and deaths (from 45 in 1990 to 184 in 2060; Fig. 3).

In comparison to the stationary population scenario, the projected population scenario predicted a total of 38.3% less varicella cases and 16.4% more HZ cases (accumulated over the study period from 2004 to 2060; Table 1). Differences in the number of hospitalizations associated with varicella across the two population scenarios to some extent reflected the difference observed for cases (–30.8%), whereas the number of deaths was higher (+13.8%) in the projected population scenario compared to the stationary population scenario. With respect to HZ, there were 24.8% more hospitalizations and 42.9% more deaths in the projected population than in the stationary population scenario.

**Table 1:** Relative impact of vaccination strategies on the predicted cumulative number of varicella and herpes zoster (HZ) cases (in the period 2004–2060) by population scenario

	Varicella cases (differences in %^a^)	HZ cases (differences in %^a^)
Stationary population		
Varicella vaccination^b^	–72.55%	+11.15%
Varicella + HZ (current live) vaccination^c^	–72.57%	+9.90%
Varicella + HZ (new subunit) vaccination^c^	–72.63%	+6.15%
Projected population		
Varicella vaccination^b^	–69.70%	+10.84%
Varicella + HZ (current live) vaccination^c^	–69.72%	+9.52%
Varicella + HZ (new subunit) vaccination^c^	–69.80%	+5.18%
Projected population with increased immigration		
Varicella vaccination^b^	–68.90%	+10.94%
Varicella + HZ (current live) vaccination^c^	–68.92%	+9.60%
Varicella + HZ (new subunit) vaccination^c^	–69.01%	+5.29%

^a^Compared to scenario without varicella and HZ vaccination

^b^Vaccination coverage 86.9%/64.1% (2004–2010 as observed)

^c^Vaccination coverage 20%

In the projected population with increased migration, slightly higher numbers of varicella and HZ cases were predicted than in the projected population not considering increased migration. Moreover, there were slightly more hospitalizations (varicella +9.7%; HZ +2.9%) and HZ-associated deaths (+0.8%). The number of varicella-associated deaths in the year 2060 increased by 46.7%, which corresponds to a rise from 6.0 absolute cases in the projected population to 8.8 in the projected population with increased migration (Fig. 2).

### Effects of varicella vaccination

In all three population scenarios, universal varicella vaccination resulted in a strong immediate decline in varicella cases up to a maximum relative reduction of 90% in 2014 when compared to corresponding scenarios without vaccination (Fig. 2, upper panel). After 2014, varicella case numbers increased to approximately one-third of the cases observed in corresponding scenarios without vaccination, mainly due to breakthrough infections. Despite the strong overall reduction in varicella case numbers, varicella case numbers increased among persons older than 9 years due to an age-shift (Additional file 1: Figure S5). The impact of vaccination is opposed by the fact that complication rates increase with age, so that the reduction in cases led to only a small overall reduction in hospitalizations and even an increase in deaths. However, it must be noted that, while deaths associated with varicella increased considerably on a relative scale, they were still very small in absolute numbers (on average below 10 deaths per year for all of Germany). In addition, the estimation of the total number of deaths associated with varicella (or HZ) was quite difficult as most deaths associated with varicella occur in multimorbid patients, where a unique definition of exact cause of death is usually not possible.

Given the assumed reduction in boosting of immunity against HZ, varicella vaccination increased the number of HZ cases by a maximum of 21% in the stationary population and 18% in both projected populations (compared to no vaccination) in the year 2026 (Fig. 3). In 2060, the reduced HZ incidence in varicella-vaccinated individuals already compensated for the reduction of boosting in all population scenarios regarding HZ case numbers, but not for hospitalizations or deaths. Nevertheless, in the long run, HZ cases, hospitalizations, and deaths were reduced by more than 50% compared to scenarios without varicella vaccination [19].

### Effects of HZ vaccination

HZ vaccination with the currently licensed live attenuated vaccine only had limited effects on the epidemiology of HZ under base case assumptions, mainly due to the low expected vaccination coverage of 20%, the short duration of protection, and the age-dependent declining efficacy. For hospitalizations and deaths associated with HZ, the relative reduction was even smaller (Fig. 3). In contrast, the new vaccine candidate, under the same coverage assumptions, was able to reduce the excess HZ cases (additional HZ cases due to varicella vaccination) by 45–52% due to higher vaccine efficacy and longer duration of protection (Table 2). While the currently licensed HZ vaccine did not compensate for the 11% excess in HZ cases (aggregated over the period 2004 to 2060) due to varicella vaccination even with 100% coverage, the new HZ vaccine candidate was predicted to fully compensate for excess HZ cases at vaccination coverage rates of approximately 40% (Additional file 1: Figures S6 and S7).

**Table 2:** Relative impact of projected population scenarios on the predicted cumulative number of varicella and herpes zoster (HZ) cases (in the period 2004–2060) by vaccination scenario

	Varicella cases (differences in %^a^)		HZ cases (differences in %^a^)	
	Projected population 1^b^	Projected population 2^c^	Projected population 1^b^	Projected population 2^c^
No vaccination	–38.31%	–37.12%	+16.45%	+21.59%
Varicella vaccination	–31.90%	–28.76%	+16.12%	+21.35%
Varicella + HZ (current live) vaccination	–31.91%	–28.77%	+16.05%	+21.26%
Varicella + HZ (new subunit) vaccination	–31.95%	–28.82%	+15.39%	+20.60%

^a^Compared to the stationary population model

^b^Without short-term immigration

^c^With short-term immigration

For the purpose of comparability, age of vaccination was set for the new subunit HZ vaccine at 60 years, which has been shown to be the best age of vaccination regarding the reduction of overall HZ cases for the currently licensed vaccine. However, due to its longer duration of protection, a younger age at vaccination would be even more efficient for the new subunit vaccine since a vaccination age of 60 years would result in a lifelong protection, even under conservative estimations for protection duration. Nevertheless, for a decision on the best age of vaccination, more information about the loss of vaccine protection over time would be necessary. The relative impact of both varicella and HZ vaccination (compared to no vaccination) was similar across all population scenarios (Table 2).

### Projected future epidemiology of varicella and HZ

According to predictions from the scenario that represents the best current population dynamics and vaccination recommendations in Germany (projected population with increased migration scenario, with varicella vaccination, and without HZ vaccination), varicella cases decreased from almost 1 million in 1990 to approximately 800,000 in 2003 due to demographic changes; case numbers will then fall rapidly due to varicella vaccination and stabilize at approximately 170,000 cases from 2020 onwards (Fig. 2). HZ cases are predicted to increase from approximately 400,000 in 1990 to 640,000 in 2033 and will then slowly decrease to 530,000 cases in 2060. The minimum number of varicella cases, hospitalizations, and deaths is predicted for the year 2014 (Fig. 3). While the number of varicella cases (–82.0%) and hospitalizations (–45.8%) will be much smaller in 2060 than in 1990, this will not be the case for the number of deaths (+155.9%). HZ case numbers will peak around 2030 with the highest hospitalization and death numbers following in 2046 and 2056. All three HZ outcomes will be much higher in 2060 than in 1990.

### Combined effects of demographic changes and vaccination strategies

In order to assess the relative contribution of vaccination and demographic changes, we compared the epidemiology in the years 2003 (year before varicella vaccination) and 2060 (as predicted by the projected population model; Table 3). The number of hospitalizations for varicella and HZ are primarily driven by demographic changes, whereas the overall case numbers for varicella are mostly affected by vaccination strategies. In the short run, demographic changes and varicella vaccination lead jointly to decreasing varicella cases and hospitalizations, and to increasing HZ cases and hospitalizations. In the long run, the effects of a declining population and protection from varicella vaccination against HZ exceed that of an aging population and absence of boosting, so that demographic changes and varicella vaccination will also jointly lead to a decrease regarding HZ case numbers and hospitalizations.

**Table 3:** Comparison of burden of varicella and herpes zoster (HZ) for the years 2003 and 2060 in different vaccination scenarios in the projected population model

	Year	2003	2060	2060	2060	2060
Scenario	Varicella vaccination	no	no	yes	yes	yes
	HZ vaccination	no	no	no	current live	new subunit
Varicella	Cases	776,007	511,908	167,422	167,241	166,659
	Hospitalizations	1,752	1,192	989	987	979
	Deaths	5	6	12	12	11
HZ	Cases	449,896	484,723	474,080	466,465	429,361
	Hospitalizations	14,453	21,515	23,540	23,305	21,215
	Deaths	54	184	214	214	196

## Discussion

We analyzed the expected changes of varicella and HZ epidemiology due to the combined effects of demographic changes and vaccination against both varicella and HZ. Our results show that long-term demographic changes will be a major driver of varicella and HZ epidemiology in the next 50 years in Germany. With decreasing numbers of varicella cases and increasing numbers of HZ cases, both diseases would have the same incidence rates in 2060. Since not only HZ incidence, but also HZ hospitalization rates and case fatality rates increase substantially with age, deaths and hospitalizations associated with HZ are predicted to increase considerably. Within all three population scenarios, varicella vaccination would lead to a massive reduction of varicella cases and, when taking the boosting hypothesis into account, to a temporary increase in HZ cases. Despite the strong effects of demography on the epidemiology of varicella and HZ, our study suggests that the predicted relative impact of vaccinations was very similar across all population scenarios.

Few previous studies have analyzed the effect of changing population structures on VZV epidemiology [5, 13]. Karhunen et al. [13] implemented structural changes in the size and composition of the population, but did not take into account the resulting changes in contact patterns. The study by Marziano et al. [5] was, to our knowledge, the only one which explicitly modelled changes of contact patterns due to demographic changes over time, but did not assess how different vaccination strategies might interact with short- and long-term assumptions on population predictions. We showed that assumptions on population structures have a considerable effect on the predicted burden of disease for varicella and HZ, while they have almost no effect on vaccination impact estimates. Furthermore, migration has little effect on the epidemiology and no effect on the impact of vaccination.

Including complex dynamics, as performed herein by taking demographic changes into account, comes with the drawback that the uniqueness of the model is lost, leading to the question of which of the simplifying assumptions (e.g. stationary population vs. country-specific population structures, stable mortality rates, or changes in medical treatment) should and could be replaced by realistic projections. A specific problem of using a projected population approach is the long time frame – a period of at least 100 years is necessary to capture all positive effects of varicella vaccination on the burden of HZ. At the same time reasonable population projections for the next 100 years are difficult to obtain. In particular, in Germany, Destatis provides only population projections up to the year 2060, restricting our analysis to this period although a longer time frame would be necessary for the consideration of all positive effects of varicella vaccination. Moreover, even supposedly certain assumptions, such as the population projections for the next 5 years, may at times be challenged by unpredictable events like the increased immigration observed in Europe in 2015/2016.

Our study indicates that there are only small differences in model predictions when age-standardizing results, such that a potential alternative approach could be to simply apply predictions of a stationary demographic vaccination model to predicted changes in the population age structure. However, this approach would not be more efficient since most of the complexity and uncertainty in the model is attributable to boosting and demographic changes; therefore, adding vaccination to the model, which is nevertheless necessary due to the dynamic process, is simple and does not increase uncertainty or limitations.

### Strengths and limitations

The present analysis is based on the best available evidence as it uses official predictions for population development and data from the pre-vaccination era for model calibration. Moreover, we were able to include information on the efficacy of an HZ vaccine candidate as well as on short-term population changes due to the migration movements in 2015/2016. The underlying model has been tested extensively with respect to parameter sensitivity for varicella and HZ in a stationary population setting [19] (chapter 12 in Additional file 1).

A major limitation of our study is the uncertainty associated with the model parameters and assumptions used in the model, in particular with regards to the boosting hypothesis, which was the driving factor of the effect of varicella vaccination on HZ epidemiology [19]. We used the widely accepted boosting mechanism based on Brisson et al. [11] and supplemented our results with a number of different sensitivity analyses by, for example, considering different assumptions about the boosting mechanism. Due to the large population size and the high prevalence of VZV infection, the multiple runs of a stochastic model will not differ considerably from the deterministic model; therefore, we decided to use a simplified deterministic modelling approach. Apart from demographic predictions, the main uncertainty in the model results from several unknown model parameters related to VZV infection (e.g., boosting hypothesis or waning rates of varicella or HZ vaccination) and cannot be solved by using random stochastic fluctuations of different realizations of the model. Since we were interested in specific effects of one of the sources of uncertainty (population development) on vaccination impact, we chose a deterministic model where all other parameters were fixed. To model demographic changes we used the geometric mean to balance changing population proportions; for simplicity and due to lack of available data we did not model changes in demographics before 1990 or potential changes over time in age-specific mixing rates due to behavioral changes like, for example, the establishment of day-care centres in Germany or the population movements after the reunification of Germany. Whereas there is plenty of information available about the epidemiology of varicella and the effects of varicella vaccination (efficacy, effectiveness, duration of protection), and some about the currently licensed HZ vaccine, information on the new HZ vaccine candidate is based on the results of two phase III clinical trials without information on long-term efficacy. VZV seroprevalence estimates were, with two exceptions, not based on studies in migrants, but were inferred using information from their countries of origin as a proxy. However, a first pilot study among immigrants in Germany in 2016 provided consistent VZV seroprevalence estimates [24]. One major limitation of our work is that most of the results of our study could not be validated using data from the post-vaccination era. The ongoing surveillance system for varicella and HZ cases in Germany started in April 2005, almost 1 year after introduction of varicella vaccination and is based on voluntary reporting, and is therefore able to measure broad trends in disease burden; no continuous reliable recording system for varicella or HZ incidence exists in Germany. In contrast, hospitalizations and deaths associated with varicella and HZ have been recorded in Germany in a reliable way since 2000. Nevertheless, the absolute number of deaths is too small to recognize reliable trends as predicted by our model. Due to reimbursement issues, there is an overall trend to higher hospitalization rates with shorter durations in Germany. This is also true for HZ, complicating the analysis of trends in hospitalization rates, which already started to increase before introduction of varicella vaccination. In most countries, just as in Germany, HZ incidence and hospitalization rates increased considerably in the last decades. In the US, for example, most studies suggest an increase of HZ incidence; however, its exact extent varies between studies. Similar to Germany, this increase occurred independently of the introduction of varicella vaccination, indicating that there must be at least one additional factor explaining the variation. Effects of demographic changes on HZ resulting from the boosting hypothesis would be expected to be weaker than those of vaccination. Therefore, even with both factors of demographic change and varicella vaccination in place, the observed patterns in HZ epidemiology could not be entirely explained. In addition, this trend is not consistent across studies focusing on hospitalization rates.

## Conclusions

In our study, we showed that analyses based on projected population scenarios predict 11–14% more HZ cases and 31–38% less varicella cases over the next 50 years compared to a stationary population scenario, irrespective of the vaccination strategy implemented. The long-term demographic changes in Germany will have a considerable effect on the epidemiology of both varicella and HZ. In contrast, short-term population changes (as observed in 2015/2016) have only minimal effects on varicella and HZ outcomes. In addition, demographic and vaccination effects seem to be almost independent from each other. A stationary population approach might therefore be sufficient if the focus of the analysis is to assess the relative impact of vaccination strategies compared to no vaccination. Nevertheless, assessing vaccination strategies in a projected population scenario will provide a more complete picture for decision-making, which is necessary for resource planning or a correct interpretation of future surveillance data [25].

## Additional file

Additional file 1: Influence of demographic changes on the impact of vaccination against varicella and herpes zoster in Germany – a mathematical modelling study (technical appendix). (DOCX 1750 kb)

## Abbreviations

Destatis: Federal Statistical Office of Germany
HZ: Herpes zoster
VZV: Varicella-zoster virus
