# Supplementary material for: Influence of demographic changes on the impact of vaccination against varicella and herpes zoster in Germany – a mathematical modelling study
Source: BMC Med. 2018 Jan 9;16:3. doi: 10.1186/s12916-017-0983-5 (PMC5761134; doi:10.1186/s12916-017-0983-5)
Supplement: Additional file 1: — Influence of demographic changes on the impact of vaccination against varicella and herpes zoster in Germany – a mathematical modelling study (technical appendix). (DOCX 1750 kb) [file 12916_2017_983_MOESM1_ESM.docx]

Additional file 1

Influence of demographic changes on the impact of vaccination against varicella and herpes zoster in Germany – a mathematical modelling study (technical appendix)

Johannes Horn, Oliver Damm, Wolfgang Greiner, Hartmut Hengel, Mirjam E Kretzschmar, Anette Siedler, Bernhard Ultsch, Felix Weidemann, Ole Wichmann, André Karch, Rafael T Mikolajczyk

Table of contents

1. Model structure of the underlying SEIR model……………………………………..…2
2. Available data for calibration……………………………………………………....…..3
3. Parameters…………………………………………………………………………...…3
4. Model fit to observed data……………………………………………………………...5
5. Effects of the demographic change on contact patterns………………………………...6
6. Age-specific effects of varicella vaccination on varicella………………………………7
7. Vaccination against herpes zoster in the projected population scenario………………...8
8. Assumptions for the projected population with increased migration…………………...9
9. Incidence and age-standardized incidence for varicella and herpes zoster……………10
10. Estimation of effectiveness of varicella vaccination…………………………………..11
11. Estimation of the duration of protection (new subunit HZ vaccine) …………………..11
12. Sensitivity analysis…………………………………………..………………………..11
13. Equations…………………………………………..………………………………….15

References………………………….………………………………………………….….16

### 1. Model structure of the underlying SEIR model (see Horn and colleagues [[1](#_ENREF_1)])

The deterministic dynamic compartmental model consists of four segments (see Figure S1):

A. Natural varicella disease (S, E, I, R; first row);

B. Varicella vaccination (V1, V2, VL);

C. Breakthrough varicella disease (SB, EB, IB, R; second row)

D. Herpes zoster (HZ) disease (SZ, IZ, RZ) and HZ vaccination (VZ);

subscripts define the type of HZ (N = natural, B = breakthrough, V = vaccine type).

Compartments for natural varicella disease occurring in the model represent the natural course of varicella disease in the absence of any protection from varicella vaccination (M=maternal immunity, S=susceptible, E=exposed(infected), I=infectious, R=resistant). In contrast, breakthrough varicella is defined as the generally milder form of varicella in individuals partly protected by varicella vaccine in second row (using prefix B for breakthrough). HZ stages are differentiated with respect to the type of primary varicella disease using subscripts (N=natural, B=breakthrough or V=vaccine breakthrough). Vaccine breakthrough is a very rare side effect of a vaccination with a live vaccine which leads to disease caused by the vaccine virus in absence of wild virus infection observed almost exclusively in immunocompromised children.


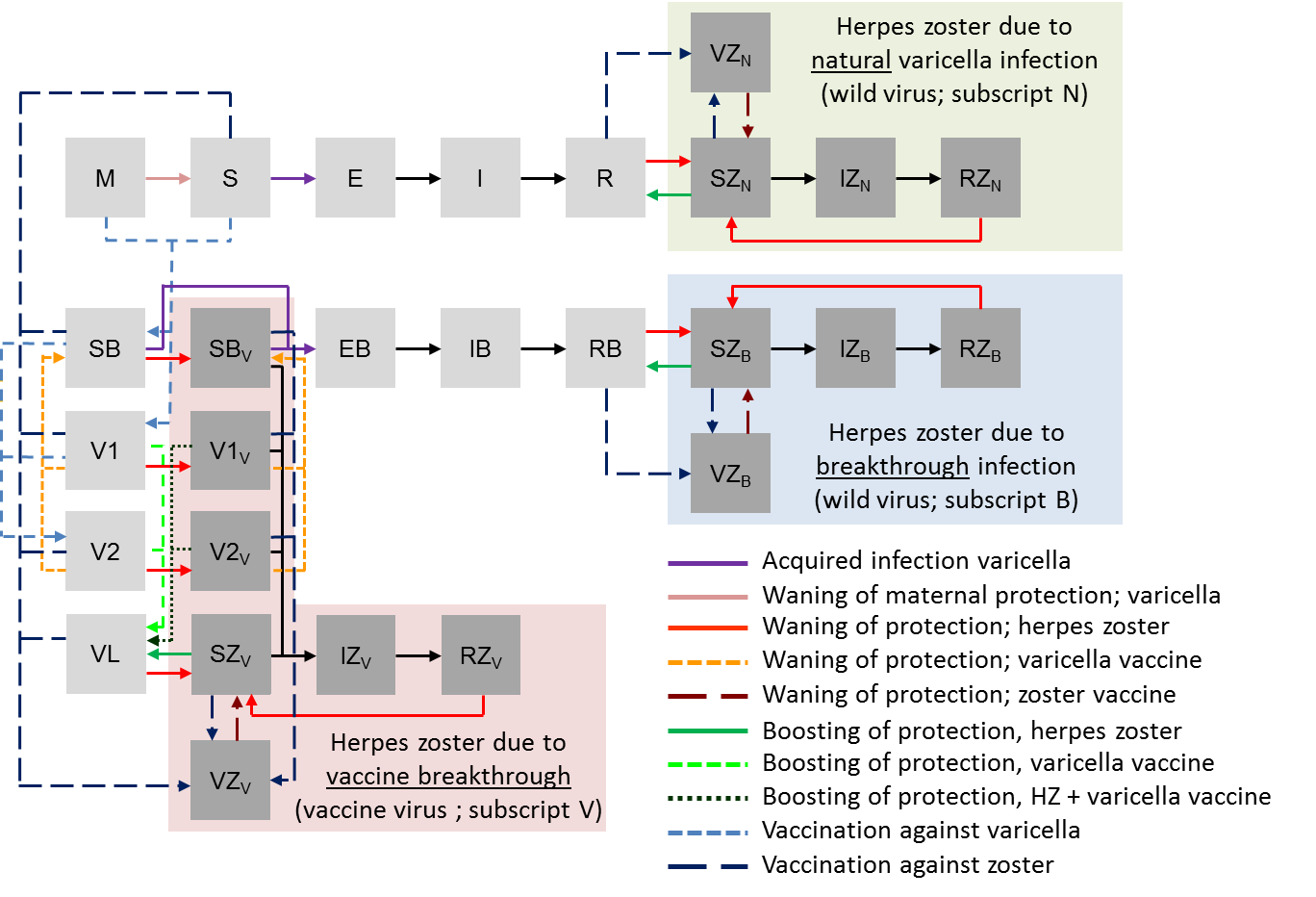


Figure S1: Model structure

M = maternal protection; S, E, I, R = susceptible, exposed (latent), infectious, resistant regarding natural varicella; SB; EB, IB, RB = susceptible, exposed, infectious, resistant regarding breakthrough varicella; SZ, IZ, RZ, VZ susceptible, infectious, resistant, vaccinated regarding herpes zoster; V1, V2 = vaccinated against varicella with 1 or 2 doses ; VL = varicella vaccine-related protection boosted to lifelong duration; subscripts of Z (ZN, ZB, ZV) = specification of zoster type due to natural varicella (N), breakthrough varicella (B) or vaccine virus (V); SBV, V1V, V2V = shortcut for SB_SZV, V1_SZV, V2_SZV defining individuals susceptible, vaccinated against varicella (with one or two doses) and susceptible to herpes zoster due to vaccine virus.

Transmission of the virus as well as boosting of protection against HZ or extension of varicella vaccine protection was modelled dynamically using contact matrices provided by the POLYMOD project. Breakthrough varicella was assumed to be half as infectious as natural varicella [[8](#_ENREF_8), [9](#_ENREF_9)] which in turn was 10 times as infectious as HZ. Depending on infectivity, contact with an infectious individual (I,IB, IZN,B,V) will lead to new varicella infections in susceptible individuals (S, SB, SB) as well as boosting of protection against HZ (SZ_N_,_B_,_V_,V1_V_,V2_V_) or boosting of varicella vaccine protection (V1,V2,V1_V_,V2_V_).

### 2. Available data for calibration

1. Seroprevalence, before the introduction of varicella vaccination in 19 age groups (different ranges; blood samples from 1995 to 1999)[[2](#_ENREF_2)].
2. Incidence of HZ in 5-year age groups for persons older than 50 years (2007 to 2008) [[3](#_ENREF_3)] and 10-year age groups for persons under 50 years (2004 to 2008)[[4](#_ENREF_4)].
3. Hospitalization and mortality rates of varicella and HZ (data 2000 to 2011) [[5](#_ENREF_5), [6](#_ENREF_6)]
4. Varicella vaccination coverage (one dose/two dose) at the age of 24 months (year 2006 to 2011) [[1](#_ENREF_1)]
5. Proportional reduction of varicella cases (data 2005 to 2012, partly published in [[7](#_ENREF_7)])

Only the variables infectivity of varicella per contact (for unvaccinated persons) and reactivation rate of herpes zoster by age and sex were directly calibrated using least sum of squares minimization. Parameters for vaccination (efficacy, primary vaccine failure, waning) were not calibrated but calculated to fit to the reported effectiveness of varicella vaccination [[10](#_ENREF_10), [11](#_ENREF_11)] and checked for validity according to data form the German varicella sentinel [[7](#_ENREF_7)]. For simplicity and to get a wider data basis, fitting was performed for the year 2003 (the year before introduction of varicella vaccination) but separately for the stable population as well as the projected population. As some of the data came from different time periods, all available data as well as the estimates for the projected population (there are no changes for the stable population model as it is in the steady state before the introduction of varicella vaccination) were checked for any signs of visible changes in the epidemiology of varicella or herpes zoster. In the projected population model incidence changed only considerably if not adjusted for age. Observed data indicate an increase of herpes zoster incidence from 2004 to 2008, which is not significant. Hospitalization and mortality rates for herpes zoster but not so much for varicella increased over the whole period of 2000 to 2011; however, there were no differences in slope between before and after vaccination indicating unspecific changes towards higher and shorter hospitalization rates as seen for many other diagnoses in Germany. Unlike in adults or teenagers, hospitalization rates for herpes zoster in children under 10 years old decreased as expected by more than half after the introduction of vaccination, which can be linked to varicella vaccination. Therefore, data after vaccination (2004 to 2011) from persons less than 10 years old was excluded for the purpose of calibration.

### 3. Parameters

Table S1 contains the most important parameters of the model.

Table S1: Parameter values used for the model

| Parameter | Base case value | Source |
| --- | --- | --- |
| **Varicella** | | |
| Duration of maternal immunity | 150 days | [[12](#_ENREF_12)] |
| Duration of latent period | 14 days | assumed according to [[8](#_ENREF_8), [9](#_ENREF_9), [13](#_ENREF_13)] |
| Duration of infectious period (unvaccinated individuals) | 7 days | [[8](#_ENREF_8), [9](#_ENREF_9), [13](#_ENREF_13)] |
| Probability of virus transmission (per contact) | 0.117 | calibrated |
| **Varicella vaccination** | | |
| Primary failure* | 4% | [[8](#_ENREF_8), [9](#_ENREF_9), [13](#_ENREF_13)] |
| Vaccine effectiveness  (1 dose / 2 doses) | 90% / 96% | calculated |
| Average duration of vaccine protection (1 dose / 2 doses) | 40 / 80 years | assumed |
| Vaccination coverage (1 dose / 2 doses) | 86.9% / 64.1% (from 2011 on) | see methods |
| Probability of boosting varicella vaccine protection (per infectious contact) | 100% | [[8](#_ENREF_8)] |
| **Varicella in vaccinated individuals** | | |
| Relative infectivity of breakthrough varicella (compared to natural varicella) | 50% | [[8](#_ENREF_8), [9](#_ENREF_9)] |
| Duration infectious period (vaccinated person) | 4.5 days | [[9](#_ENREF_9), [13](#_ENREF_13), [14](#_ENREF_14)] |
| **Herpes Zoster (HZ)** | | |
| Duration of protection against HZ after: varicella infection (any type), HZ episode, boosting of protection against HZ, varicella vaccination (vaccine virus) | 20 years | Assumed [[8](#_ENREF_8), [9](#_ENREF_9), [13](#_ENREF_13)] |
| Probability of boosting the protection against HZ (per infectious contact) | 100% | Assumed [[8](#_ENREF_8), [9](#_ENREF_9)] |
| Reactivation rate of VZV leading to HZ (age and sex specific) | 0.9%-5.3% | calibrated |
| Relative reactivation rate of VZV in individuals vaccinated against varicella (compared to unvaccinated individuals)  a) After breakthrough varicella b) Reactivation of vaccine virus | a) 47.6417%  b) 12.8614% | calculated based on [[15](#_ENREF_15)] |
| Duration of infectious period (HZ) | 28 days | [[12](#_ENREF_12), [13](#_ENREF_13)] |
| Relative infectivity of HZ (compared to natural varicella) | 0.05 | [[16](#_ENREF_16)] |
| **HZ vaccination** |  |  |
| Vaccination coverage (live HZ vaccine) | 20% | assumed |
| Vaccine efficacy (live HZ vaccine) | 18-70% (age-dependent) | [[17-19](#_ENREF_17)] |
| Duration of protection (live HZ vaccine) | 12 years | [[20](#_ENREF_20), [21](#_ENREF_21)] |
| Best age of vaccination | 60 years | Horn and colleagues [[1](#_ENREF_1)] |
| Vaccination coverage (new subunit HZ vaccine) | 20% | assumed |
| Vaccine efficacy (new subunit HZ vaccine) | 97.2% | [[22](#_ENREF_22)] |
| Duration of vaccine protection (new subunit HZ vaccine) | 56 years | see chapter 11 |

* Immediate vaccine failure (main reason no seroconversion) not to be confused with waning of vaccine protection

### 4. Model fit to observed data

*Serology varicella*


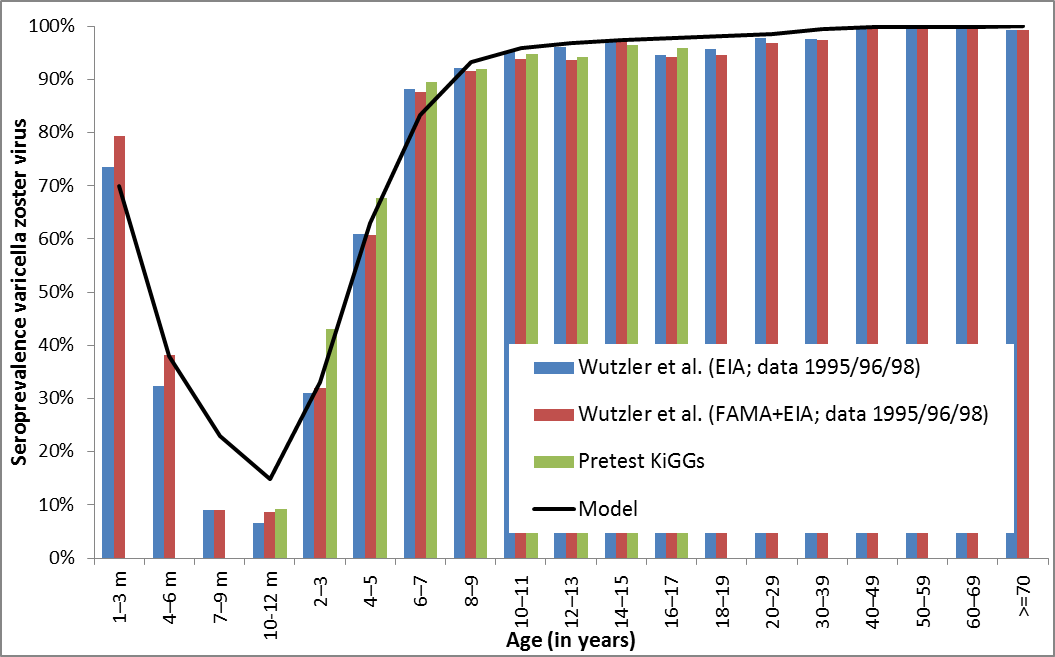


Figure S2: Seroprevalence of varicella in Germany, by age (data [[2](#_ENREF_2), [23](#_ENREF_23)])

*Herpes Zoster*


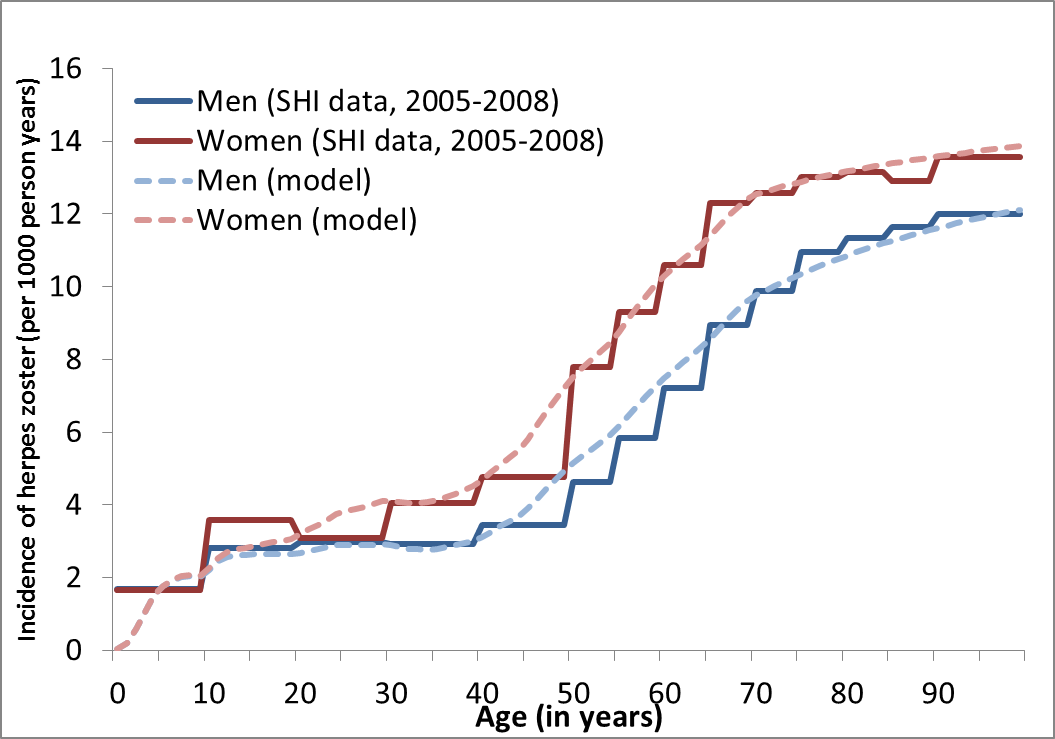


Figure S3: Incidence of herpes zoster in Germany, by sex and age (data [[3](#_ENREF_3), [4](#_ENREF_4)]; SHI = statutory health insurance)

### Unfortunately, there are not much more data about varicella or herpes zoster cases available in Germany. Including the data already mentioned there are two data sources about seroprevalence of varicella[[2](#_ENREF_2), [23](#_ENREF_23)] in the pre-vaccination era as well as publications evaluating incidence of herpes zoster with the help of health insurance data reporting almost identical age and sex-specific HZ incidence rates; all of them monitor the same time period (2004 to 2009) after vaccination [[3](#_ENREF_3), [4](#_ENREF_4), [24](#_ENREF_24), [25](#_ENREF_25)].

The general problem is a lack of incidence data before the introduction of varicella vaccination. The only publication with varicella and herpes zoster incidence data before introduction of varicella vaccination [26] recorded in outpatient medical practices in a small town (1992 - 1993) suffers from methodological problems. Physician-reported varicella incidence was only half of the rates necessary to fit observed seroprevalence data. Moreover, reported HZ incidence was less than half of the rate reported in later studies. Most available data after vaccination is not processed, analysed or published. Current surveillance started after introduction of vaccination; health insurance data in Germany is only consistent since 2004, the year of introduction of varicella vaccination.

Nevertheless annual hospitalization rates and mortality rates were recorded for varicella and herpes zoster since 2000, thus including time-periods before as well as after the introduction of varicella vaccination. However, these data are prone to time-dependent biases and provide inconsistent results. For example, the two sources of mortality rates for herpes zoster show different trends (A: principal diagnoses of all persons which die in hospital (strong increase), B: reason of death according to death certificate (constant)). In addition, similar to other countries there is a strong continuous increase of herpes zoster hospitalization rates across all recorded years (2000 - 2015) which does neither fit to an possible impact of varicella vaccination nor to demographic changes, indicating that there must be additional causes.

### 5. Effects of the demographic change on contact patterns

Due to demographic changes, the number of children is expected to be almost halved whereas the number of older persons more than doubles until 2060. Due to the balancing of contacts using the square root, the contacts from older persons to children almost halve while contacts from children to older person double (see Figure S4). Middle-aged persons have slightly less contacts with children and more contact s with the elderly.


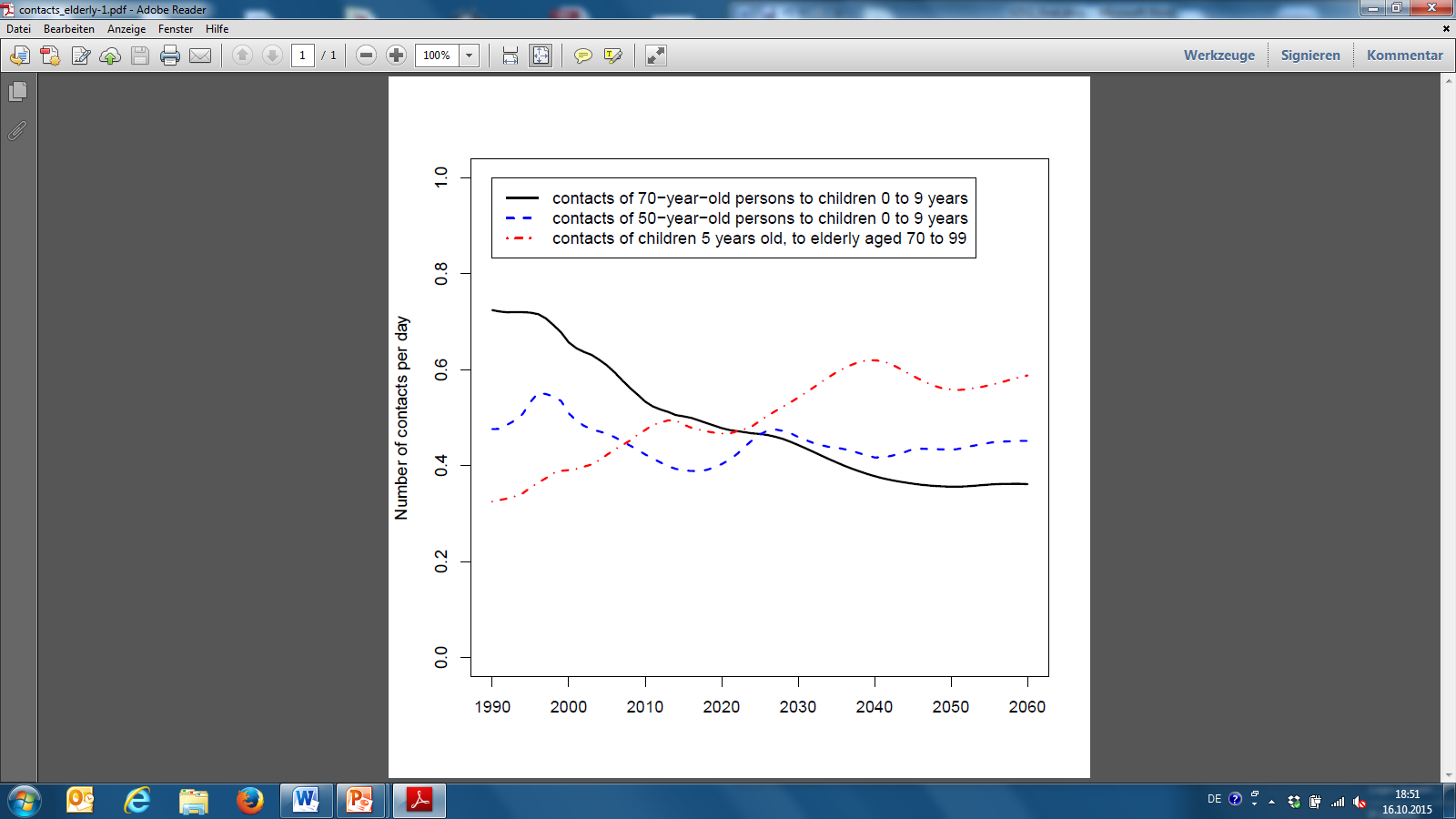


Figure S4: Changes of number of contacts per day over time in three exemplary age groups

It has to be noted that our model reacts differently to demographic changes than the model proposed by Marziano et al. [[27](#_ENREF_27)]. In our model the number of contacts are adjusted according to the square route of the ratio of the numbers of persons in both age-classes using square routes, whereas in Marziano et al. the contacts of each age-class are balanced according to the number of persons in the age-class of the contact.

If, as an example, we look for simplicity reasons only at three hypothetical age-classes:

1. Young children
2. Their parents
3. Their grandparents

Let us assume that in case of demographic changes the proportion of children decreases (for example by 40%) whereas the proportion of grandparents increase compared to a stable population by a factor of 2. The proportion of the total population in the age group of young parents stays about the same. Using the approach of Marziano et al. the contact rates would change when compared to the stationary population by a factor of:

|  | Contacts from a | | |
| --- | --- | --- | --- |
|  | Child | Parent | Grandparent |
| Contacts to |  |  |  |
| Children | 0.6 | 0.6 | 0.6 |
| Parents | 1 | 1 | 1 |
| Grandparents | 2 | 2 | 2 |

In contrast in case of our model contacts would change compared to a stationary population by:

|  | Contacts from a | | |
| --- | --- | --- | --- |
|  | Child | Parent | Grandparent |
| Contacts to |  |  |  |
| Children | 1 | $\sqrt{0.6}\sim0.77$ | $\sqrt{0.6/2}\sim0.55$ |
| Parents | $\sqrt{1/0.6}\sim1.29$ | 1 | $\sqrt{0.5}\sim0.71$ |
| Grandparents | $\sqrt{2/0.6}\sim1.83$ | $\sqrt{2}\sim1.41$ | 1 |

The approach by Marzaino et al. is motivated by the idea of random mixing of persons. Therefore if the frequency of one age-group changes over time, all contacts to this age-group change by this respective factor whereas the contact behaviour of the respective age-groups does not change at all. Our approach is motivated by the idea of balancing desired contact rates according to wishes of both contact partners by correcting resulting inequalities with the geometric mean. Compared to Marziano, our approach predicts smaller changes due to demographic effects. For example, in our model the age-specific varicella incidence will not change considerably due to demographic changes since the number of contacts is usually highest to persons of the same age and these will not change in our approach. With Marziano’s approach, contacts of young children with each other would be halved resulting in an age-shift of the epidemiology of varicella towards older age-groups. Boosting contacts for the elderly (to children) would however decrease in both models at almost the same magnitude.

Indirectly associated with the question of contact patterns are the boosting assumptions. Most previous VZV models followed the simplified approach of Brisson et al. in which boosting is only possible if protection against HZ has already waned [9] and in which boosting leads to a temporal immunity against HZ. In Karhunen et al. and Guzzetta et al., boosting events do not lead to immunity, but reduce the reactivation rate. To which extent the reactivation rate is reduced depends in Karhunen et al. on age and time since last contact with VZV, in Guzzetta et al. also on the total number of boosting events of each individual so far [28, 29]. For simplicity all models use in their base case analyses the assumption that all contacts which would lead to an infection in case of a susceptible person will lead to a boosting event for a person susceptible to HZ. Furthermore, as most of the calibration data comes from the pre-varicella vaccination era, the estimated baseline reactivation rate is estimated based on these model assumptions.

### 6. Age-specific effects of varicella vaccination on varicella

There is only a small difference in the effect of varicella vaccination on age-specific incidence of varicella between the different population scenarios. The observed differences in case numbers are mainly due to changes in the population age structure as the age-specific incidences are almost identical (see Figure S5)

**
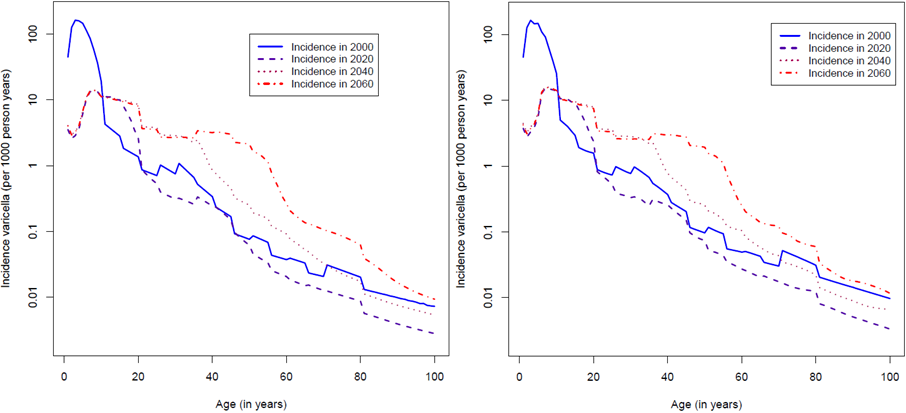
**

Figure S5: Age-specific effect of varicella vaccination on varicella incidence in the *stable population* (left) and *projected population* model (right). Note the log scaling of the y-axes.

Note: In the model, contact patterns are modelled according to age-stratified contact data from the POLYMOD survey. Whenever a person moves into the next age-class, contact frequency and patterns are adapted which can lead to a considerable different risk of varicella infection.

### 7. Vaccination against herpes zoster in the *projected population* scenario

In the *projected population* scenario, it is even more evident than in the *stationary population* scenario that HZ vaccination at the age of 60 years is the most effective way to reduce HZ incidence. This should not be confused with cost-effectiveness as the total number of vaccinated persons changes with age at vaccination (see Figure S6).


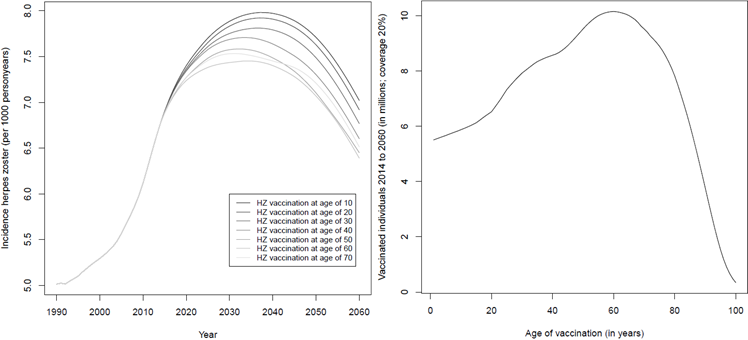


Figure S6: Effect of new HZ vaccine on HZ cases by age of vaccination (left); the total number of HZ vaccinated individuals in the time perdiod 2014-2060, when the age of HZ vaccination is varied over the whole age range from 0 to 100 (right)

### 8. Assumptions for the projected population with increased migration

The *projected population with increased migration* was introduced to account for the short-term migration as observed in Germany in 2015/2016. In this period, the extent of immigration to Germany was substantially higher than already accounted for in the demographic predictions. Moreover, in contrast to previous years, when most migrants came from other European countries with similar VZV seroprevalence as Germany, in 2015 most migrants came from countries with substantially lower VZV seroprevalence. For example, while in Germany only about 3% of all adults are susceptible to varicella, in most countries of origin for migrants coming to Germany in 2015, about 10% of adults were susceptible to varicella (Table S2).

The *projected population with increased migration* is based on the *projected population* obtained from the Federal Statistical Office of Germany. However, it is assumed that an additional one million migrants came to Germany in 2015 and from 2016 to 2025 this yearly additional number of migrants is assumed to drop by 100,000 per year, until it reaches zero in 2026. These migrants’ countries of origin are assumed to follow the distribution of the top countries of origin of asylum seekers in Germany in 2015 (see Table S2). Due to restrictions of published data, no country-specific age or sex distributions were available so that the overall age distribution was applied to all countries of origin.

We applied the same method (using the same POLYMOD baseline matrices and balancing discrepancies with the geometric mean) to get contact matrices for migrants as for the rest of the population. In the first year after arrival their contact network was restricted to other migrants except for one additional contact per day to a random person in the original population. After the first year, it was assumed that only the proportion of migrants according to the official protection rate specific for the country of origin (persons either are accepted asylum seekers/refugees, were granted temporary protection or deportation ban) remained in Germany. These individuals were from then on included in the compartmental model capturing the rest of the German population.

To obtain seroprevalence estimates by country of origin for children and adults, a short literature review was performed (using the terms “seroprevalence”, “varicella”, “refugees”, and the respective country of origin). Information was found for Syria (only adults) [[28](#_ENREF_28)], Iraq [[29](#_ENREF_29), [30](#_ENREF_30)], Kosovo (only children) [[29](#_ENREF_29)], and Eritrea [[31](#_ENREF_31)]. In case no data were available, information was retrieved for neighbouring countries; in case of Syria data from Turkey [[32](#_ENREF_32), [33](#_ENREF_33)] and in case of Afghanistan and Pakistan data from Iran [[34-36](#_ENREF_34)] were used. In case of Albania, Kosovo, Serbia, and Macedonia, the mean estimates of data from Greece, Turkey, Kosovo, and former Yugoslavia were used for children [[29](#_ENREF_29), [32](#_ENREF_32), [33](#_ENREF_33), [37](#_ENREF_37)]; for adults only data from Turkey were available and were used.

Table S2: Distribution of asylum seekers in Germany by country of origin, age and sex in 2015 (according to the Federal Office for Migration and Refugees)

| Distribution by country of origin | | | | | Distribution by age and sex | | |
| --- | --- | --- | --- | --- | --- | --- | --- |
| Country | Asylum  seekers | Seroprevalence (children) | Seroprevalence  (adults) | Protection rate (2014)* | Age | Men | Women |
| Syria | 162510 | 72.05% | 90.82% | 89.35% | 0 to 15 | 47466 | 39032 |
| Albania | 54762 | 67.48% | 91.33% | 2.23% | 16 to 17 | 11910 | 3152 |
| Kosovo | 37095 | 67.48% | 91.33% | 1.08% | 18 to 24 | 65505 | 15925 |
| Afghanistan | 31902 | 46.17% | 82.89% | 46.70% | 25 to 29 | 38813 | 12414 |
| Iraq | 31379 | 69.45% | 96.52% | 73.95% | 30 to 34 | 25274 | 10480 |
| Serbia | 26945 | 67.48% | 91.33% | 0.20% | 35 to 39 | 16089 | 7723 |
| Eritrea | 14131 | 36.90% | 88.04% | 55.24% | 40 to 44 | 10484 | 5004 |
| Macedonia | 10990 | 67.48% | 91.33% | 0.26% | 45 to 49 | 6346 | 3209 |
| Pakistan | 8472 | 46.17% | 82.89% | 18.62% | 50 to 54 | 3493 | 2091 |
|  | | | | | 55 to 59 | 1768 | 1434 |
|  |  |  |  |  | 60 to 64 | 943 | 825 |
|  |  |  |  |  | 65++ | 859 | 987 |

* no data for 2015 available; protection rate includes accepted asylum seekers/refugees, granted temporary protection, and deportation ban

### 9. Incidence and age-standardized incidence for varicella and herpes zoster

For all analyses, relative vaccination effects and effects of demographic changes measured based on incidence rates were consistent with those measured based on absolute case numbers as the change in total population size over time is almost negligible. Differences between the stationary population scenario and the projected population scenarios were mostly removed when using standardized incidence rates instead of absolute case numbers or observed incidence rates (Figure S7). The increased short-term migration had only temporary effects on varicella epidemiology but also minor long-term effects on HZ. Directly after the immigration impulse, there was a small, temporally limited increase of varicella cases followed by a general increase of hospitalizations and deaths associated with varicella. In addition, there was a slight increase of HZ incidence, hospitalizations, and mortality rates. Both effects can be attributed to the lower VZV seroprevalence in migrants, especially in adults when compared to the autochthonous German population. Changing the year, the extent of migration, or adding a second period of increased migration did not change this finding qualitatively but almost linearly shifts the effects in time, changes the extent of the effects, or adds a second similar effect of migration respectively.


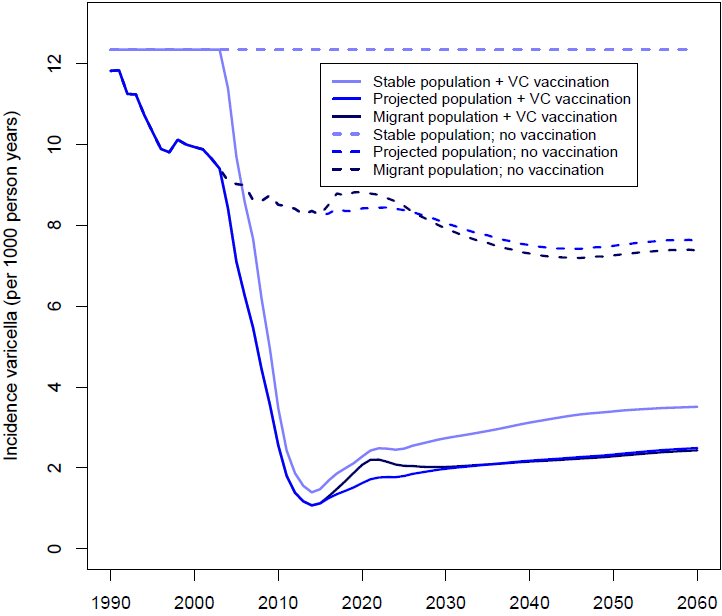

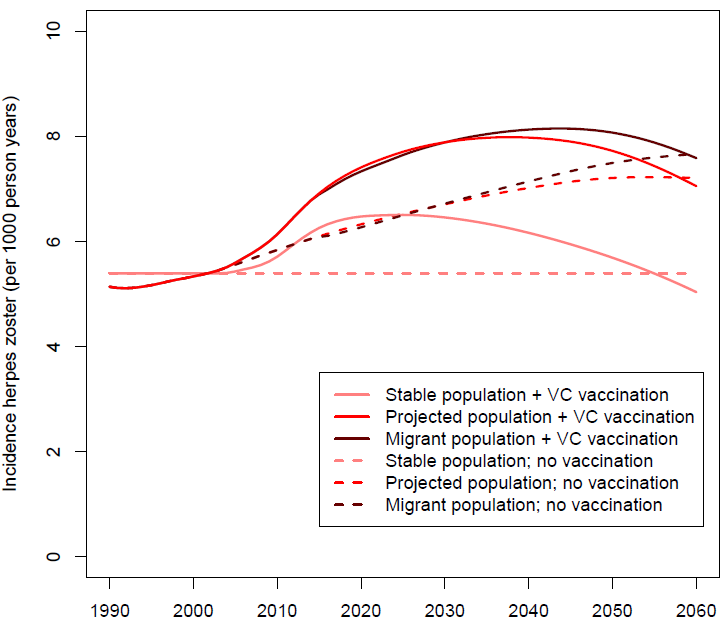

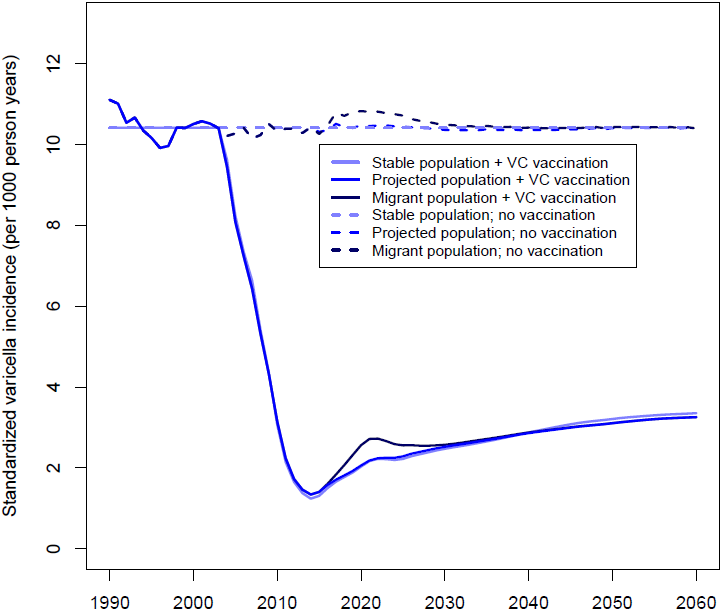

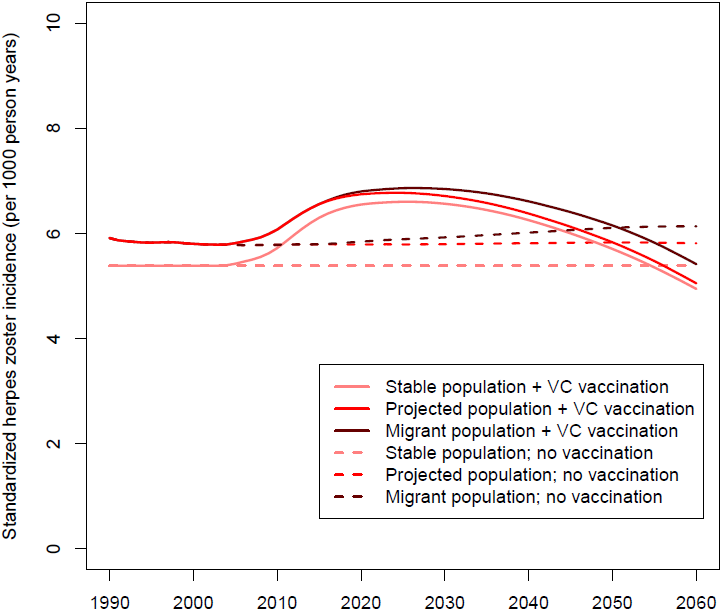


Figure S7: Incidence (top) and age-standardized* incidence (bottom) for varicella (left) and herpes zoster (right) by model population

*Age standardized according to European standard population 2013

### 10. Estimation of effectiveness of varicella vaccination

Similar to other modelling studies we incorporated vaccine effectiveness against all varicella cases by taking into account both overall effectiveness as well as effectiveness against moderate or severe varicella cases in particular [[8](#_ENREF_8), [9](#_ENREF_9)]. We did that by assuming in the first step a primary vaccine failure rate of (4%) which reflects the seroconversion rates as well as 1 minus the vaccine effectiveness (96%) against severe cases reported by Seward and colleagues [[11](#_ENREF_11)]. Any person in the model with a primary vaccine failure would be considered as not vaccinated regarding severity of disease, whereas varicella in any vaccinated person without primary vaccine failure would almost always be mild. Under these assumptions, vaccine effectiveness (1 dose schedule) in persons without primary vaccine failure was calculated to be 90% considering the reported vaccine effectiveness of Bayer and colleagues of 73% [[10](#_ENREF_10)] and the one of Seward and colleagues of 81% [[11](#_ENREF_11)] as well as the reported average follow-up time. As there is neither a study reporting a decline of vaccine effectiveness over time nor a study with a follow up period of more than ten years, waning rates could only be assumed. Moreover, there was no information about vaccine effectiveness for a two dose schedule except for the study of Kuter and colleagues [[38](#_ENREF_38)] which reported vaccine effectiveness rates which are not in line with data from observed outbreak investigations. After calibration based on the studies mentioned above, a review by Marin and colleagues [[39](#_ENREF_39)] was published reporting a vaccine effectiveness for a 1-dose schedule identical to the one reported by Seward and colleagues (81%) against any varicella cases; at the same time they report a higher effectiveness (98%) against moderate or severe varicella cases. In addition, the study contains also vaccine effectiveness data for a two dose vaccination schedule against any varicella cases of 92%; taking into account reported follow up time and assumed primary failure rates this fits exactly to the assumptions used in this modelling study.

### 11. Estimation of the duration of protection (new subunit HZ vaccine)

We calculated the duration of protection for the new subunit HZ vaccine as described by Lal and colleagues 2015 using [[22](#_ENREF_22)]:

(I) Mean duration of follow up 3.2 years

(II) Observed overall vaccine efficacy for age 60 years and older: 97.2% => mean vaccine efficacy over 3.2 years is 97.2%

Assumption for HZ incidence: constant within 3.2 years follow-up (conservative since incidence increases with age)

Assumption for observed vaccine failure: All observed cases of breakthrough infection are due to waning of protection; none due to primary vaccine failure (initial failure, main reason no seroconversion). This assumption is conservative, as potential effects of primary vaccine failure would result in weaker effect of waning and therefore longer duration of vaccine protection.

Assumption for waning: exponential

⇒ mean vaccine efficacy = $\frac{\int_{0}^{3.2} e^{-x/d}dx}{3.2}=0.972$ (d = duration vaccine efficacy in years)

⇒ $d-d* e^{-\frac{3.2}{d}}-0.972*3.2=0$ ⇒ solving numerically $d \approx56.07$years

### 12. Sensitivity analyses

HZ vaccination coverage

Independently of the applied vaccine and the population scenario the effects of HZ vaccination coverage on the epidemiology of HZ are almost linear. Each one percent increase in vaccination coverage of the live attenuated vaccine reduced the cumulative number of HZ cases (over the period 2004 to 2060) by 0.06-0.07% (dependent on the population scenario used); in case of the new HZ vaccine candidate a one percent increase in vaccination coverage led to a reduction of 0.26-0.29% in cumulative HZ case numbers (Figure S8 and S9). Hence, the currently licensed HZ vaccine cannot compensate the 11% excess in HZ cases (aggregated over the period 2004 to 2060) due to varicella vaccination even with 100% coverage; the new HZ vaccine candidate is predicted to fully compensate excess HZ cases at vaccination coverage rates of about 40%.

**
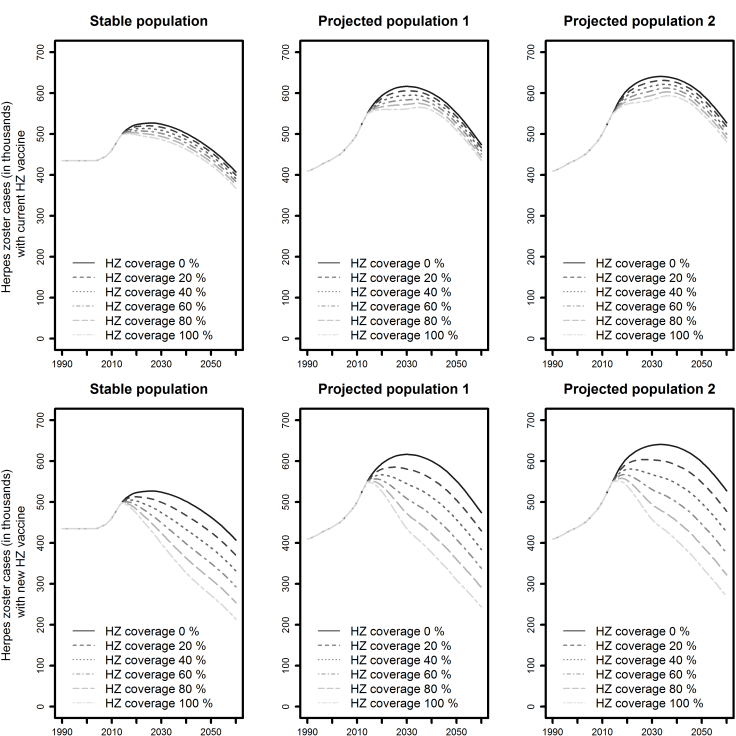
**

Figure S8: Impact of HZ vaccine coverage on the epidemiology of HZ (current live and new subunit vaccine)

The main reason of the potentially higher impact of the new vaccine is not its higher effectiveness but its longer duration of protection. While the current vaccine with an age at vaccination of 60 years only reduces HZ incidence in the age ranges from 60 to 70 years, the new HZ vaccine affects all age classes over 60 years (Figure S9). As age-classes over 70 years are decisive for the overall number of hospitalizations and deaths, HZ vaccination at an age of 60 years with the current HZ Vaccine has almost no effect on these outcomes, whereas the long term impact of the new subunit vaccine on hospitalizations and deaths is apart from a delay similar to that on HZ cases (see manuscript Figure 3).

**
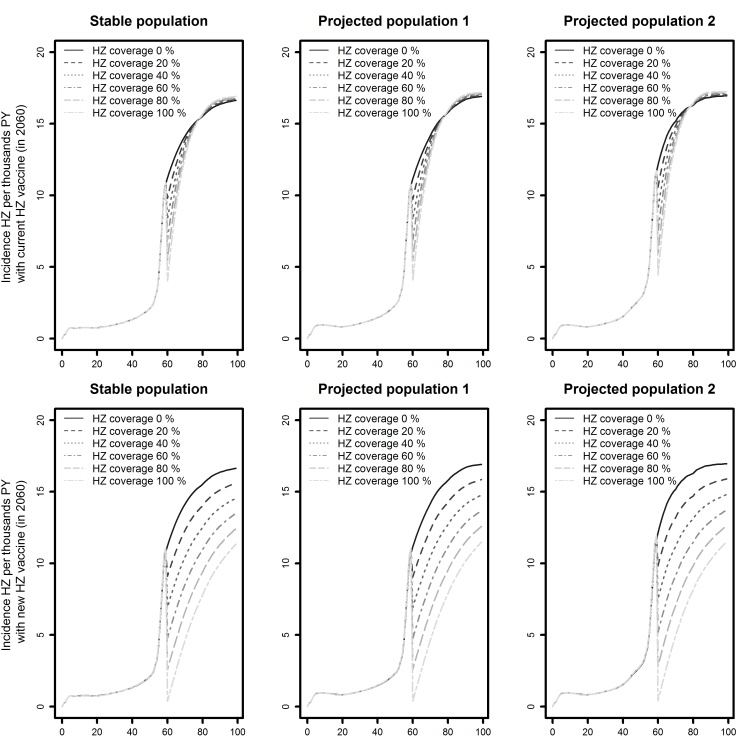
**

Figure S9: Impact of HZ vaccine coverage on age-specific incidence of HZ in 2060 (current live and new subunit vaccine)

Time period of increased migration

In a sensitivity analysis we varied the time period of increased migration between 2015 (base case), 2030, and 2045. Impact of varicella or HZ vaccination on respective incidence rates was almost unaffected.

Table S3: Relative impact of vaccination strategies on the predicted cumulative number of varicella and herpes zoster (HZ) cases (in the period 2004-2060) by time period of increased migration (in reference to table 1 of the main manuscript)

|  | Varicella cases (differences in %*) | HZ cases (differences in %*) |
| --- | --- | --- |
| Projected pop. increased immigration 2015 |  |  |
| Varicella vaccination^±^ | -68.90% | +10.94% |
| Varicella + HZ (current live) vaccination^#^ | -68.92% | +9.60% |
| Varicella + HZ (new subunit) vaccination^#^ | -69.01% | +5.29% |
| Projected pop. increased immigration 2030 |  |  |
| Varicella vaccination^±^ | -68.85% | +10.81% |
| Varicella + HZ (current live) vaccination^#^ | -68.87% | +9.49% |
| Varicella + HZ (new subunit) vaccination^#^ | -68.96% | +5.19% |
| Projected pop. increased immigration 2045 |  |  |
| Varicella vaccination^±^ | -68.75% | +10.73% |
| Varicella + HZ (current live) vaccination^#^ | -68.77% | +9.41% |
| Varicella + HZ (new subunit) vaccination^#^ | -69.86% | +5.09% |

* compared to scenario without varicella and HZ vaccination

± vaccination coverage 86.9%/64.1% (2004-2010 as observed)

# vaccination coverage 20%

Table S4: Relative impact of time period of increased migration on the predicted cumulative number of cases varicella and herpes zoster (HZ) cases (in the period 2004-2060) by vaccination scenario migration (in reference to table 2 of the main manuscript)

|  | Varicella cases  (differences in %*) | | | HZ cases  (differences in %*) | | |
| --- | --- | --- | --- | --- | --- | --- |
| Increased migration in (year) | 2015 | 2030 | 2045 | 2015 | 2030 | 2045 |
| No vaccination | -37.12% | -37.17% | -37.28% | +21.59% | +20.26% | +19.53% |
| Varicella vaccination | -28.76% | -28.69% | -28.60% | +21.35% | +19.88% | +19.06% |
| Varicella + HZ (current live) vaccination | -28.77% | -28.70% | -28.61% | +21.26% | +19.81% | +18.98% |
| Varicella + HZ (new subunit) vaccination | -28.82% | -28.75% | -28.65% | +20.60% | +19.18% | +18.33% |

* compared to the stationary population model

± without short-term immigration

# with short-term immigration

Other sensitivity analyses

While there is no real uncertainty regarding the natural history of varicella infection, we performed a number of sensitivity analyses focusing on vaccine parameters and the role of boosting (HZ, varicella vaccine protection) [[1](#_ENREF_1)]. In these analyses, varicella breakthrough cases had only a considerable impact on overall burden of disease if all of the following applied:.

1. Duration of varicella vaccine protection is not lifelong
2. Contact with varicella has only minor effects on varicella vaccine protection (no boosting)
3. Varicella breakthrough cases are considerable more severe in adults than in children.

Similarly, reduced boosting against HZ led in these analyses only to a considerable temporal increase of HZ incidence if both of the following applied:

1. More than 50% of all contacts with varicella result into boosting against HZ
2. Duration of protection against HZ from a boosting event lasts at least 10 years

The varied parameters in these sensitivity analyses affected all population scenarios in virtually the same way so that differences between population scenarios were not affected by parameter uncertainty.

### 13. Equations

$$\frac{dM\left( a,t \right)}{dt}=M\left( a,t \right)-b M\left( a,t \right)+v\left( a,t \right)*M(a,t)$$

$$\frac{dS\left( a,t \right)}{dt}=S\left( a,t \right)+b M(a,t)-(\lambda\left( a,t \right)+v\left( a,t \right))*S\left( a,t \right)$$

$$\frac{dE\left( a,t \right)}{dt}=E\left( a,t \right)+\lambda\left( a,t \right)*S\left( a,t \right)-c E(a,t)$$

$$\frac{dI\left( a,t \right)}{dt}=I\left( a,t \right)+c E\left( a,t \right)-d I(a,t)$$

$$\frac{dR\left( a,t \right)}{dt}=R\left( a,t \right)+d I\left( a,t \right)+e \lambda\left( a,t \right)*SZ\left( a,t \right)-f R\left( a,t \right)$$

$$\frac{dSZN\left( a,t \right)}{dt}=SZN\left( a,t \right)-e \lambda\left( a,t \right)*SZN\left( a,t \right)-g\left( a,s \right) SZN\left( a,t \right)+h RZN\left( a,t \right)+f R(a,t)$$

$$\frac{dIZN\left( a,t \right)}{dt}=IZN\left( a,t \right)+g\left( a,s \right) SZN\left( a,t \right)-i IZN(a,t)$$

$$\frac{dRZN\left( a,t \right)}{dt}=RZN\left( a,t \right)+i IZN(a,t)-h RZN(a,t)$$

$$\frac{dSB\left( a,t \right)}{dt}=v1e*v\left( a,t \right)*\left( M\left( a,t \right)+S\left( a,t \right) \right)-\left( f+\lambda\left( a,t \right) \right) SB\left( a,t \right)+vw1 V1\left( a,t \right)+vw2 V2(a,t)$$

$$\frac{dSBV\left( a,t \right)}{dt}=SBV\left( a,t \right)- \lambda\left( a,t \right) SBV\left( a,t \right)+f*\mathrm{SB}\left( a,t \right)-k g\left( a,s \right) SBV\left( a,t \right)+vw1 V1V\left( a,t \right)+vw2 V2V(a,t)$$

$$\frac{dV1\left( a,t \right)}{dt}=V1\left( a,t \right)+\left( 1-v1e \right)*v\left( a,t \right)*\left( M\left( a,t \right)+S\left( a,t \right) \right)-(f+vw1+\lambda\left( a,t \right)) V1(a,t)$$

$$\frac{dV2\left( a,t \right)}{dt}=V2\left( a,t \right)+\left( 1-v2e \right)*v2\left( a,t \right)*\left( SB\left( a,t \right)+V1\left( a,t \right) \right)-(f+vw2+\lambda\left( a,t \right)) V2(a,t)$$

$$\frac{dVL\left( a,t \right)}{dt}=VL\left( a,t \right)+\lambda\left( a,t \right)\left( V1\left( a,t \right)+V2\left( a,t \right)+V1V\left( a,t \right)+V2V\left( a,t \right)+SZV(a,t) \right)-f VL(a,t)$$

$$\frac{dEB\left( a,t \right)}{dt}=EB\left( a,t \right)+\lambda\left( a,t \right)*(SB\left( a,t \right)+SBV(a,t))-c EB(a,t)$$

$$\frac{dIB\left( a,t \right)}{dt}=IB\left( a,t \right)+c EB\left( a,t \right)-d2 IB(a,t)$$

$$\frac{dRB\left( a,t \right)}{dt}=RB\left( a,t \right)+d2 IB\left( a,t \right)+e\lambda\left( a,t \right)*SZB\left( a,t \right)-fRB\left( a,t \right)$$

$$\frac{dSZB\left( a,t \right)}{dt}=SZB\left( a,t \right)-e\lambda\left( a,t \right)*SZB\left( a,t \right)-j g\left( a,s \right) SZB\left( a,t \right)+h RZB\left( a,t \right)+f RB(a,t)$$

$$\frac{dIZB\left( a,t \right)}{dt}=IZB\left( a,t \right)+j g\left( a,s \right) SZB\left( a,t \right)-i IZB(a,t)$$

$$\frac{dRZB\left( a,t \right)}{dt}=RZB\left( a,t \right)+i IZB(a,t)-h RZB(a,t)$$

$$\frac{dV1V\left( a,t \right)}{dt}=V1V\left( a,t \right)+f V1\left( a,t \right)-(k g\left( a,s \right)+\lambda\left( a,t \right)+vw1) V1V(a,t)$$

$$\frac{dV2V\left( a,t \right)}{dt}=V2\left( a,t \right)+f V2\left( a,t \right)-\left( k g\left( a,s \right)+\lambda\left( a,t \right)+vw2 \right) V2V(a,t)$$

$$\frac{dSZV\left( a,t \right)}{dt}=SZV\left( a,t \right)-e\lambda\left( a,t \right)*SZV\left( a,t \right)-k g\left( a,s \right) SZV\left( a,t \right)+h RZV\left( a,t \right)+f VL(a,t)$$

$$\frac{dIZV\left( a,t \right)}{dt}=IZV\left( a,t \right)+k g\left( a,s \right) (SBV\left( a,t \right)+V1V\left( a,t \right)+V2V(a,t)+SZV\left( a,t \right))-i IZV(a,t)$$

$$\frac{dRZV\left( a,t \right)}{dt}=RZV\left( a,t \right)+i IZV(a,t)-h RZV(a,t)$$

Force of infection:

$$\lambda\left( a,t \right)= \sum_{a^{,}} \beta(a,a^{,},t) (x I\left( a^{,},t \right)+y IB\left( a^{,},t \right)+z(IZN(a^{,},t)+IZB(a^{,},t)+IZV(a^{,},t)))$$

## References:

1. Horn J, Karch A, Damm O, Kretzschmar ME, Siedler A, Ultsch B, Weidemann F, Wichmann O, Hengel H, Greiner W *et al*: **Current and future effects of varicella and herpes zoster vaccination in Germany - insights from a mathematical model in a country with universal varicella vaccination**. *Human vaccines & immunotherapeutics* 2016:0.

2. Wutzler P, Farber I, Wagenpfeil S, Bisanz H, Tischer A: **Seroprevalence of varicella-zoster virus in the German population**. *Vaccine* 2001, **20**(1-2):121-124.

3. Ultsch B, Siedler A, Rieck T, Reinhold T, Krause G, Wichmann O: **Herpes zoster in Germany: quantifying the burden of disease**. *BMC infectious diseases* 2011, **11**:173.

4. Ultsch B, Koster I, Reinhold T, Siedler A, Krause G, Icks A, Schubert I, Wichmann O: **Epidemiology and cost of herpes zoster and postherpetic neuralgia in Germany**. *The European journal of health economics : HEPAC : health economics in prevention and care* 2012.

5. Siedler A, Dettmann M: **Hospitalization with varicella and shingles before and after introduction of childhood varicella vaccination in Germany**. *Human vaccines & immunotherapeutics* 2014, **10**(12):3594-3600.

6. Statistisches Bundesamt: **GENESIS Online-Datenbank**. In*.*; 2015.

7. Siedler A, Arndt U: **Impact of the routine varicella vaccination programme on varicella epidemiology in Germany**. *Euro surveillance : bulletin Europeen sur les maladies transmissibles = European communicable disease bulletin* 2010, **15**(13).

8. van Hoek AJ, Melegaro A, Zagheni E, Edmunds WJ, Gay N: **Modelling the impact of a combined varicella and zoster vaccination programme on the epidemiology of varicella zoster virus in England**. *Vaccine* 2011, **29**(13):2411-2420.

9. Brisson M, Edmunds WJ, Gay NJ, Law B, De Serres G: **Modelling the impact of immunization on the epidemiology of varicella zoster virus**. *Epidemiol Infect* 2000, **125**(3):651-669.

10. Bayer O, Heininger U, Heiligensetzer C, von Kries R: **Metaanalysis of vaccine effectiveness in varicella outbreaks**. *Vaccine* 2007, **25**(37-38):6655-6660.

11. Seward JF, Marin M, Vazquez M: **Varicella vaccine effectiveness in the US vaccination program: a review**. *The Journal of infectious diseases* 2008, **197 Suppl 2**:S82-89.

12. Garnett GP, Grenfell BT: **The epidemiology of varicella-zoster virus infections: the influence of varicella on the prevalence of herpes zoster**. *Epidemiol Infect* 1992, **108**(3):513-528.

13. Schuette MC, Hethcote HW: **Modeling the effects of varicella vaccination programs on the incidence of chickenpox and shingles**. *Bulletin of mathematical biology* 1999, **61**(6):1031-1064.

14. Izurieta HS, Strebel PM, Blake PA: **Postlicensure effectiveness of varicella vaccine during an outbreak in a child care center**. *JAMA : the journal of the American Medical Association* 1997, **278**(18):1495-1499.

15. Weinmann S, Chun C, Schmid DS, Roberts M, Vandermeer M, Riedlinger K, Bialek SR, Marin M: **Incidence and clinical characteristics of herpes zoster among children in the varicella vaccine era, 2005-2009**. *The Journal of infectious diseases* 2013, **208**(11):1859-1868.

16. Poletti P, Melegaro A, Ajelli M, Del Fava E, Guzzetta G, Faustini L, Scalia Tomba G, Lopalco P, Rizzo C, Merler S *et al*: **Perspectives on the impact of varicella immunization on herpes zoster. A model-based evaluation from three European countries**. *PloS one* 2013, **8**(4):e60732.

17. Oxman MN, Levin MJ, Johnson GR, Schmader KE, Straus SE, Gelb LD, Arbeit RD, Simberkoff MS, Gershon AA, Davis LE *et al*: **A vaccine to prevent herpes zoster and postherpetic neuralgia in older adults**. *The New England journal of medicine* 2005, **352**(22):2271-2284.

18. Oxman MN, Levin MJ, Shingles Prevention Study G: **Vaccination against Herpes Zoster and Postherpetic Neuralgia**. *The Journal of infectious diseases* 2008, **197 Suppl 2**:S228-236.

19. Schmader KE, Levin MJ, Gnann JW, Jr., McNeil SA, Vesikari T, Betts RF, Keay S, Stek JE, Bundick ND, Su SC *et al*: **Efficacy, safety, and tolerability of herpes zoster vaccine in persons aged 50-59 years**. *Clinical infectious diseases : an official publication of the Infectious Diseases Society of America* 2012, **54**(7):922-928.

20. de Boer PT, Pouwels KB, Cox JM, Hak E, Wilschut JC, Postma MJ: **Cost-effectiveness of vaccination of the elderly against herpes zoster in The Netherlands**. *Vaccine* 2013, **31**(9):1276-1283.

21. Ultsch B, Weidemann F, Reinhold T, Siedler A, Krause G, Wichmann O: **Health economic evaluation of vaccination strategies for the prevention of herpes zoster and postherpetic neuralgia in Germany**. *BMC health services research* 2013, **13**:359.

22. Lal H, Cunningham AL, Godeaux O, Chlibek R, Diez-Domingo J, Hwang SJ, Levin MJ, McElhaney JE, Poder A, Puig-Barbera J *et al*: **Efficacy of an adjuvanted herpes zoster subunit vaccine in older adults**. *The New England journal of medicine* 2015, **372**(22):2087-2096.

23. **Zur Seroprävalenz gegen Varizella-Zoster-Virus und zur Verlässlichkeit anamnestischer Angaben**. *Epidemiologisches Bulletin* 2003, **43**.

24. Schiffner-Rohe J, Jow S, Lilie HM, Koster I, Schubert I: **[Herpes zoster in Germany. A retrospective analyse of SHL data]**. *MMW Fortschritte der Medizin* 2010, **151 Suppl 4**:193-197.

25. Hillebrand K, Bricout H, Schulze-Rath R, Schink T, Garbe E: **Incidence of herpes zoster and its complications in Germany, 2005-2009**. *The Journal of infection* 2015, **70**(2):178-186.

26. Paul E, Thiel T: **[Epidemiology of varicella zoster infection. Results of a prospective study in the Ansbach area]**. *Der Hautarzt; Zeitschrift fur Dermatologie, Venerologie, und verwandte Gebiete* 1996, **47**(8):604-609.

27. Marziano V, Poletti P, Guzzetta G, Ajelli M, Manfredi P, Merler S: **The impact of demographic changes on the epidemiology of herpes zoster: Spain as a case study**. *Proceedings Biological sciences* 2015, **282**(1804):20142509.

28. Barah F: **Prevalence of herpes simplex types 1 and 2, varicella zoster virus, cytomegalovirus, immunoglobulin G antibodies among female university students in Syria**. *Saudi medical journal* 2012, **33**(9):990-994.

29. Figueira M, Christiansen D, Barnett ED: **Cost-effectiveness of serotesting compared with universal immunization for varicella in refugee children from six geographic regions**. *Journal of travel medicine* 2003, **10**(4):203-207.

30. Leung J, Lopez A, Mitchell T, Weinberg M, Lee D, Thieme M, Schmid DS, Bialek SR: **Seroprevalence of varicella-zoster virus in five US-bound refugee populations**. *Journal of immigrant and minority health / Center for Minority Public Health* 2015, **17**(1):310-313.

31. Ghebrekidan H, Ruden U, Cox S, Wahren B, Grandien M: **Prevalence of herpes simplex virus types 1 and 2, cytomegalovirus, and varicella-zoster virus infections in Eritrea**. *Journal of clinical virology : the official publication of the Pan American Society for Clinical Virology* 1999, **12**(1):53-64.

32. Kanra G, Tezcan S, Badur S, Turkish National Study T: **Varicella seroprevalence in a random sample of the Turkish population**. *Vaccine* 2002, **20**(9-10):1425-1428.

33. Gurgoze MK, Yilmaz E, Godekmerdan A, Akca Z, Dogan Y, Akarsu S, Aygun AD: **Seroprevalence of mumps, varicella and rubella antibodies in children 1-16 years of age in eastern Turkey**. *The Turkish journal of pediatrics* 2006, **48**(3):185-188.

34. Allami A, Mohammadi N: **Varicella immunity in Iran: an age-stratified systematic review and meta-analysis**. *Iranian journal of microbiology* 2014, **6**(6):372-381.

35. Sharifi Z, Emadi Ghanjin S: **The Seroepidemiology of Varicella Zoster Virus (VZV) in Different Age Groups in Tehran, Iran**. *Iranian journal of allergy, asthma, and immunology* 2005, **4**(2):95-98.

36. Allami A, Mohammadi N, Najar A: **Seroepidemiology of Varicella and value of self-reported history of Varicella infection in Iranian medical students**. *International journal of occupational medicine and environmental health* 2014, **27**(2):304-313.

37. Kavaliotis J, Petridou S, Karabaxoglou D: **How reliable is the history of chickenpox? Varicella serology among children up to 14 years of age**. *International journal of infectious diseases : IJID : official publication of the International Society for Infectious Diseases* 2003, **7**(4):274-277.

38. Kuter B, Matthews H, Shinefield H, Black S, Dennehy P, Watson B, Reisinger K, Kim LL, Lupinacci L, Hartzel J *et al*: **Ten year follow-up of healthy children who received one or two injections of varicella vaccine**. *The Pediatric infectious disease journal* 2004, **23**(2):132-137.

39. Marin M, Marti M, Kambhampati A, Jeram SM, Seward JF: **Global Varicella Vaccine Effectiveness: A Meta-analysis**. *Pediatrics* 2016, **137**(3):e20153741.
